# Supplementary material for: 1894 revisited: Cross-education of skilled muscular control in women and the importance of representation
Source: PLoS One. 2022 Mar 17;17(3):e0264686. doi: 10.1371/journal.pone.0264686 (PMC8929574; doi:10.1371/journal.pone.0264686)
Supplement: Attachment — Submitted filename: Response to reviewers.docx [file pone.0264686.s001.docx]

Dear editor,

1. Please ensure that your manuscript meets PLOS ONE's style requirements, including those for file naming. The PLOS ONE style templates can be found at

https://journals.plos.org/plosone/s/file?id=wjVg/PLOSOne_formatting_sample_main_body.pdf and

[https://journals.plos.org/plosone/s/file?id=ba62/PLOSOne_formatting_sample_title_authors_affiliations.pdf](https://urldefense.com/v3/__https:/journals.plos.org/plosone/s/file?id=ba62*PLOSOne_formatting_sample_title_authors_affiliations.pdf__;Lw!!Dq0X2DkFhyF93HkjWTBQKhk!Hdpx_MBtW5o0xm_vOd0DL0OpaubntSW1psHV5ZYRjm_XtAYnkPepCxGYfs2q28uygqbO7g$)

**Authors’ response: the document has been updated per the guidelines in the link above**

2. In your Methods section, please provide a justification for the sample size used in your study, including any relevant power calculations (if applicable).

**Authors’ response: We have added a justification of our sample size, which was based on previous investigations and reports in our lab.**

3. Thank you for stating the following in the Competing Interests section:

"The authors have declared that no competing interests exist."

We note that one or more of the authors are employed by a commercial company: Zanshin Consulting, Inc.

**Authors’ response: Zanshin Consulting Inc is the company of E. Paul Zehr who is President and sole proprietor. This company purchased the games used in the research. In order to simplify we have deleted an affiliation and mention this instead in the acknowledgements.**

3.1. Please provide an amended Funding Statement declaring this commercial affiliation, as well as a statement regarding the Role of Funders in your study. If the funding organization did not play a role in the study design, data collection and analysis, decision to publish, or preparation of the manuscript and only provided financial support in the form of authors' salaries and/or research materials, please review your statements relating to the author contributions, and ensure you have specifically and accurately indicated the role(s) that these authors had in your study. You can update author roles in the Author Contributions section of the online submission form.

Please also include the following statement within your amended Funding Statement.

“The funder provided support in the form of salaries for authors [insert relevant initials], but did not have any additional role in the study design, data collection and analysis, decision to publish, or preparation of the manuscript. The specific roles of these authors are articulated in the ‘author contributions’ section.”

If your commercial affiliation did play a role in your study, please state and explain this role within your updated Funding Statement.

**Authors’ response: “The funder provided support in the form of salaries for authors [insert relevant initials], but did not have any additional role in the study design, data collection and analysis, decision to publish, or preparation of the manuscript. The specific roles of these authors are articulated in the ‘author contributions’ section.”**

3.2. Please also provide an updated Competing Interests Statement declaring this commercial affiliation along with any other relevant declarations relating to employment, consultancy, patents, products in development, or marketed products, etc.

Within your Competing Interests Statement, please confirm that this commercial affiliation does not alter your adherence to all PLOS ONE policies on sharing data and materials by including the following statement: "This does not alter our adherence to  PLOS ONE policies on sharing data and materials.” (as detailed online in our guide for authors [http://journals.plos.org/plosone/s/competing-interests](about:blank)) . If this adherence statement is not accurate and there are restrictions on sharing of data and/or materials, please state these. Please note that we cannot proceed with consideration of your article until this information has been declared.

**Authors’ response: This is no longer applicable. Please see above.**

Please include both an updated Funding Statement and Competing Interests Statement in your cover letter. We will change the online submission form on your behalf.

Please know it is PLOS ONE policy for corresponding authors to declare, on behalf of all authors, all potential competing interests for the purposes of transparency. PLOS defines a competing interest as anything that interferes with, or could reasonably be perceived as interfering with, the full and objective presentation, peer review, editorial decision-making, or publication of research or non-research articles submitted to one of the journals. Competing interests can be financial or non-financial, professional, or personal. Competing interests can arise in relationship to an organization or another person. Please follow this link to our website for more details on competing interests: [http://journals.plos.org/plosone/s/competing-interests](about:blank)

**Authors’ response: This is no longer applicable. Please see above.**

4. We note that you have indicated that data from this study are available upon request. PLOS only allows data to be available upon request if there are legal or ethical restrictions on sharing data publicly. For more information on unacceptable data access restrictions, please see http://journals.plos.org/plosone/s/data-availability#loc-unacceptable-data-access-restrictions.

In your revised cover letter, please address the following prompts:

a) If there are ethical or legal restrictions on sharing a de-identified data set, please explain them in detail (e.g., data contain potentially sensitive information, data are owned by a third-party organization, etc.) and who has imposed them (e.g., an ethics committee). Please also provide contact information for a data access committee, ethics committee, or other institutional body to which data requests may be sent.

b) If there are no restrictions, please upload the minimal anonymized data set necessary to replicate your study findings as either Supporting Information files or to a stable, public repository and provide us with the relevant URLs, DOIs, or accession numbers. For a list of acceptable repositories, please see http://journals.plos.org/plosone/s/data-availability#loc-recommended-repositories.

**Authors’ response: We have added our data to OSF (DOI 10.17605/OSF.IO/ZFYEX – https://osf.io/zfyex/)**

We will update your Data Availability statement on your behalf to reflect the information you provide.

5. PLOS requires an ORCID iD for the corresponding author in Editorial Manager on papers submitted after December 6th, 2016. Please ensure that you have an ORCID iD and that it is validated in Editorial Manager. To do this, go to ‘Update my Information’ (in the upper left-hand corner of the main menu), and click on the Fetch/Validate link next to the ORCID field. This will take you to the ORCID site and allow you to create a new iD or authenticate a pre-existing iD in Editorial Manager. Please see the following video for instructions on linking an ORCID iD to your Editorial Manager account: https://www.youtube.com/watch?v=_xcclfuvtxQ

**Authors’ response:** [**https://orcid.org/0000-0002-1749-2924**](https://orcid.org/0000-0002-1749-2924)

**Comments to the Author**

**Reviewer #1:**

This manuscript by Pearcey et al. examines the cross-education of muscular control in terms of behavioral and neural parameters. The authors claimed both trained and untrained arms showed immense improvements in behavioral parameters, and a reduction in H-reflex.

The aim of the manuscript is interesting, but I felt the methodology was inappropriate.

Main concerns
Perez et al. (ref. 26) showed "the H-max/M-max ratio were depressed after repetition of the visuo-motor skill task and returned to baseline after 10 min". Therefore, the timing of H-reflex measurement is important for your research. It should be clearly stated in the manuscript. (I think it's better to measure the H-max/M-max ratio before and just after the game in Pre and Post -tests.)

If H-reflex was measured 10 min or more after the game, the reduction in H-reflex should be nonspecific change (i.e. not related to specific task). This is quite surprising. Why a small number of repetition of the game changed the H-reflex despite the fact that we perform skilled movements in our daily life (e.g., typing, writing, cooking, video game, playing music instruments, ...).

**Authors’ response: We appreciate the concern of the reviewer, but the work of Perez et al. was conducted to show short-term plasticity in spinal reflexes in response to a single session of visuomotor training. Indeed, in the case of Perez et al. the plasticity did not remain beyond 10 minutes, but one would not expect a single bout of training to cause long-lasting plasticity. On the contrary, in the current experiment, participants performed training multiple times per session, multiple times per week for multiple weeks. This repetitive training is what results in neuroplasticity that can persist, as is the case with operant conditioning, locomotor training, strength training, and other forms of training. In this case fine motor control was trained, and reduced spinal reflex excitability has been shown in folks who are trained for fine motor control (see Nielsen J, Crone C, Hultborn H. H-reflexes are smaller in dancers from The Royal Danish Ballet than in well-trained athletes. Eur J Appl Physiol Occup Physiol. 1993;66(2):116-21. doi: 10.1007/BF01427051. PMID: 8472692.).**

H-reflex was measured from the FCR. The function of the FCR is wrist flexion, wrist abduction, and forearm pronation. I guess the wrist joint was almost fixed during the game. That is, a precise activation control for the FCR are not needed during the game. I suppose the muscles whose activation timing and intensities need to be controlled precisely during the game are extensors and flexors of fingers, elbow, and shoulder. I don't think the H-reflex measurement from the FCR is appropriate for the purpose of this study.

**Authors’ response: We thank the reviewer for their concerns, but methodological constraints limit our ability to elicit reliable H-reflexes from the distal hand muscles or muscles acting across the shoulder/elbow, and since the FCR is still involved in fine control/movement of the wrist during the game, we chose to include it as a probe for overall spinal reflex excitability. It remains for future researchers to further explore other muscles and other measures.**

H-max/M-max ratio was not statistically different between pre and post for the entire group (n=9). Discussion must be made on this result. The results obtained from a small number of subjects can easily change.

**Authors’ response:**

**We feel the issues with H-reflex measures, including our explicit statement that effects are modest, is clearly articulated in the results and discussion. We would like to reply more effectively to the reviewer based on the last sentence but don’t understand. “Can easily change” what?**

A "general trend for performance to improve" during three pre-tests must not be ignored. The differences in both time to completion and # of errors between Pre3 and Post were small especially for the untrained arm (Fig. 2A and 2B). If Pre4 were performed for the untrained arm, the results of Pre4 may be similar to the results actually obtained in Post judging from "a general trend for performance to improve". That is, it is not clear whether shorter time to completion and smaller number of errors obtained in Post were resulted from the cross-education effect or repetition of the test. It is a fatal problem that the present study did not provide evidence to deny this expectation.

**Authors’ response:**

**We are not completely certain we follow the reviewers main point? This is a training intervention where repeated exposure may lead to plastic adaptation and enhancement of skill. As such, it does remain possible that pre assessments were producing some skill learning. If so, then our evidence of changes due to 5 weeks of training is actually underrepresenting the true change. That is, any changes to pre make changes in post harder to find. So, we suggest it’s actually not a problem and instead makes our outcomes more conservative.**

Generally, brain activities during unimanual action using dominant hand and nondominant hand are asymmetric. Thus it is important to analyze the results from right arm training group and those from left arm training group separately.

**Authors’ response: We do not think it is necessary to analyze subjects based on handedness since 1) there is only one left-hand dominant subject, 2) our group showed improvement overall, and 3) all subjects followed the same trend of improvement.**

I don't think Operation® Iron Man 2 is sufficient tool to evaluate skill for the following two reasons.
1. The buzzer sound startle the subjects, which may result in movement delay. After hearing the buzzer sound repeatedly, they will not be startled by the buzzer sound. That is, familiarity to the buzzer sound would contribute to shorten the time to completion.
2. Whether a certain amount of movement error during pulling out a plastic object buzzes or not depends on the hole because all small holes have different size and shape. The extent of movement error during every pulling out motion should be measured. Counting the buzzer sound is not enough to evaluate movement error.

**Authors’ response: Overall, we disagree and believe that the game is sufficient to provide a proxy for motor skill learning. People learn to complete the game faster and with less error, which means it by definition is a way to evaluate skill. Also, it is a faithful reproduction of the original 1894 study where a buzzer sounded when errors were made. Nevertheless, we have added a statement to acknowledge that a reduction in startle may have contributed to decreased time to completion.**

Minor concerns
Abstract
L34 "the subjects" should be used instead of "women".

**Authors’ response: We disagree. The subjects are women, and therefore “women” can be used here. This is especially relevant since we are highlighting the need to study women.**

L35 Please explain the movement while playing the game instead of the name of the game.

**Authors’ response: Our preference is to keep abstract details brief. We have added details about the game in the main text of the methods.**

L35-37 No H-max/M-max ratio reduction was found for nine subjects. （I guess）no correlation was found between H-max/M-max ratio reduction and behavioral parameters.

**Authors’ response: Hmax/Mmax ratios are a gross test of overall H-reflex excitability that is not typically sensitive to slight changes due to training. Hence, the recruitment curves are more sensitive to small changes in smaller populations of motor units within the reflex pathway.**

L39 No results gave evidence to support the claim "highlight the need to focus on female participants".

**Authors’ response: We disagree. The results show that cross-education occurs in a women only sample. Women-only samples are rarely studied in the cross education field, and therefore the statement stands. We are actually rather stunned to read that the reviewer does not realize how understudied women have been in this area.**

Introduction
A "generalized motor program" presented by Lashley (1942) , and the following studies are worth considering in your research.

**Authors’ response: Thanks for the suggestion, but we believe that there are many caveats with the notion of a generalized motor program that make it irrelevant in this case. In particular, there are issues with storage and how the CNS deals with feedback during movement that are not considered within the generalized motor program framework (see central contributions to motor control in Motor Control and Learning, eds Schmidt, Lee, Winstein, Wulf and Zelaznik).**

L80
Perez et al. (ref. 26) showed "the H-max/M-max ratio were depressed after repetition of the visuo-motor skill task and returned to baseline after 10 min".

**Authors’ response: We agree that this was found which is why we use the words “during acquisition of a novel fine motor skill” indicating that there is a brief period during/immediately after training where H-reflex is reduced. We have added some context.**

L106
If you are interested in gender effect, you should include both female and male subjects.

**Authors’ response: We were not interested in gender effects but rather the cross-education of skill in women, which we have shown. Again, the reviewer seems to not understand the base issue we are trying to address. To help with this we have added clearer language in the abstract especially.**

L122
Why did you include one left-handed subject?

**Authors’ response: we did not have exclusion criteria based on handedness.**

L132-133
Judging from this sentence, H-reflex was measured three times (Pre1, Pre2, and Pre3). Why did you show the results of movement and neural parameters differently?（Fig. 2 and Fig. 3）

**Authors’ response: We have clarified that the pre-test recruitment curve variables were normalized and combined across pre-tests (i.e. 40x3 = 120 stimuli) and compared vs post.**

L137
No results of ECR, BB, and TB were shown.

**Authors’ response: It is standard practice in our lab to monitor and record activity of heteronymous muscles while recording H-reflexes to ensure remote activity does not influence our measures (please see Zehr 2002 – Considerations for use of the Hoffmann reflex in exercise studies)**

L152
Did you use a fixed order for training session at home and in the lab? (an example of a fixed order: {lab, home, home},{lab, home, home}, ...; an example of a non-fixed order: {lab, home, home}, {home, lab, home}, ...)

**Authors’ response: A fixed order was used, but this order differed between subjects in order to accommodate participants in the lab.**

L152-153
Total number of pulling out movement (55 = 11*5) should be shown clearly. It is difficult to understand the meaning of # of Errors in Fig. 2B.

**Authors’ response: We disagree. We have quantified errors based on buzzer sounds.**

L168
The effect of test on untrained arm cannot be ignored.

**Authors’ response: The effects of three tests performed 5 weeks prior to the post test is unlikely to be a major contributor, rather there is likely training induced cross-education of skill.**

L254
I don't agree this interpretation of the result. A statistical test using only Pre1, Pre2, and Pre3 would show whether behavioral parameters were constant or not among three pre-tests.

**Authors’ response: We will have to agree to disagree with the reviewer here. This is based on our comments above and our prior (and ongoing) extensive use of multiple baseline control procedures in intervention studies.**

L298-299
This isn't in consistent with Fig. 3F.

**Authors’ response: We apologize, but we don’t understand what is intended by this comment?**

L330-332
No results support this statement because only female subjects participated in this study.

**Authors’ response: The results support the inclusion of women-only samples since we have shown that cross-education of skill occurs in a women-only sample. We encourage others to study women-only samples as well.**

It is useless to compare % of skill improvements and % of strength improvements in previous studies.

**Authors’ response: We believe it is a reasonable comparison in the context it is used. In fact, relative performance assessment as a ratio or percentage is one of the most commonly used procedures to compare across studies and participants. How else would one compare strength to skill adaptation if not a relative change?**

The discussion is too short for the main topic of the study.

**Authors’ response: We do not agree but if the reviewer has specific recommendations on what to discuss in more detail, we are keen to listen. However, we don’t understand the suggestion to make the discussion longer because it is too short?**

The kinematics and muscle activity during gameplay, and enough sample size are essential to the purpose of this study.

**Authors’ response: As stated in our section on “important considerations,” we did not measure kinematics or muscle activity during gameplay, so we cannot provide this. In addition, we discuss the limitations associated with our small sample in this section, yet we have now given a rationale of our sample size, which has been added in text.**

**“The sample size was determined based on prior related work in the laboratory using similar designs and outcome measures, which were sufficient to achieve significant cross-education effects of strength training with moderate effect sizes (Barss et al 2018 doi: 10.1152/japplphysiol.00390.2017, Sun et al 2018 doi: 10.1007/s00221-018-5275-6).”**

**Reviewer #2:**

This study has investigated the inter-manual transfer of motor skills using a real gaming task. The authors demonstrated that the skill learned by one hand was substantially transferred to the skill of another hand. Furthermore, the skill acquisition was likely to be accompanied by the reduction of H-reflex induced in the FCR muscle. This is a significant study showing the presence of cross-education of motor skills in a realistic situation, but I would like to raise several points that the authors need to consider for the improvement.

**Authors’ response: Thanks for the constructive feedback, which we believe helped improve the quality of the manuscript.**

Major points
1) The information about the task was insufficient. The authors need to describe the details of the game and task more clearly. Which muscles and fingers did the subjects mainly use? What were the size of the objects, the holes, and the tweezers?

**Authors’ response: We have added some more details about the game. In particular, we briefly described that participants were not instructed how to hold the tweezers, but the most common way was to hold the tweezers between the tips of the thumb and index finger with support from the middle finger. Objects ranged in size with graspable regions ranging from 1-4mm. The tweezers were 5.4cmm in length with a 7mm aperture. The holes ranged in size, but the narrowest region was 16mm.**

2) The reduction of H-reflex induced by the practice is an interesting observation. As the authors already mentioned in the manuscript, there were several limitations, like the small number of subjects in which the reflex could be measured. However, I believe that further analysis would help examine if the reduction of H-reflex was actually related to the improvement of the performance. It would be interesting to see the correlation between the amount of improvement of performance and the amount of H-reflex reduction. I am very interested in the amount of reduction of H-reflex was correlated between both hands.

**Authors’ response: Although these aspects are interesting to explore, the small sample size severely limits our ability to run correlational/regression analyses. In addition, we have no metric of overall improvement of performance, which complicates an answer these questions (i.e. improvement in speed vs errors). We think that the change in the reflex excitability is a minor side story and have tried to downplay this finding, particularly in response to another reviewers concerns.**

Minor points
Figure 2: There must be a trade-off (speed-accuracy trade-off) between the “time to completion” and the errors. The scatter plot (e.g., x-axis is time to completion and y-axis is errors) could help the readers recognize how the practice changed the performance.

**Authors’ response: We are unsure what the reviewer thinks the scatter below adds to the story. Is this what is suggested?**

Figure 4: The current figure is not informative. Adding the recruitment curves would be helpful.

**Authors’ response: We do not agree with the reviewer and prefer Figure 4 as it is. It currently shows the relative amplitudes and shapes of waveforms (i.e. M-wave and H-reflexes) along with the latencies of the responses. True raw data is beneficial to ensure replicability in the future.**

**Reviewer #3:**

The paper by Pearcey and colleagues explores an interesting concept and is uniquely framed with reference to findings reported over 125 years ago. The phenomenon of cross-education has potential relevance to neurorehabilitation and musculoskeletal rehabilitation and the inclusion of a female-only sample addresses gaps in the current literature. The study demonstrates that unilateral training of a task with the dominant limb leads to bilateral improvement in task performance as measured by time to completion and the number of errors committed. Reductions in spinal excitability are also observed. The paper is generally well-written, but I would ask the authors to consider the following points:

**Authors’ response: Thanks for the helpful suggestions, which we think help improved the quality of the manuscript.**

1. One of the study hypotheses focuses on spinal reflex excitability, but as the Authors highlight in the introduction, transcallosal processes are likely at play. Indeed, the recent Delphi Consensus statement on cross education (Manca et al. 2021) supports the view that interhemispheric inhibition is the mechanism most likely to be mediating the phenomenon. The Authors double-down on the interhemispheric focus by introducing concepts related to stroke recovery in the present paper, where interhemispheric disinhibition is a likely contributor to motor impairment. Given the that the likely mechanisms at play are cortical in origin, it isn’t clear as to why the Authors chose to focus on measures of spinal excitability rather than cortical excitability to probe the neurophysiological underpinnings related to cross-education in muscular control. The experimental approach and hypothesis don’t quite align with putative mechanisms for the cross-education effect described by the Authors.

**Authors’ response: We certainly agree that cortical mechanisms are major factors mediating cross-education. However, cortical output must arrive at and pass through the spinal cord to be expressed as motor activity. Thus, spinal adaptations are also relevant to study. We also suggest that our study was not intended as a major mechanistic investigation.**

In lines 76-80, the use of spinal excitability assessment in the present study is justified by questioning whether there is equivalence between modulation of spinal circuitry after training for muscle control as there appears to be in muscle power. But if the primary mechanism for the phenomenon in the context of muscle power is cortically mediated, why not focus on probing whether muscular control is mediated by the same mechanism? Perhaps more justification is required in the introduction to align with the spinal reflex excitability hypothesis as postulated by the Authors.

**Authors’ response: The measurement of spinal excitability was meant to be an additional probe to examine whether this type of training influences neural circuits within the spinal cord. The main message remains that *cross-education* of *skill* occurs in *women*. A change in spinal excitability should not take away from this message, but rather provide some additional insights about what might contribute to this finding. We have made an attempt to de-emphasize the importance of the reflex findings.**

2. By having testing and training take place using the same device, there is no evidence as to whether there is a transfer effect to other tasks. While it could be argued that ‘transfer’ is what is being examined in terms of skill development from one limb to it’s untrained contralateral homologue, the functional relevance in a clinical context would require that therapeutic benefits be transferred to other tasks. While I agree that there is potential clinical utility to this approach, I wonder whether the Authors have considered this form of transfer? Perhaps the statements related to clinical relevance should be tempered or have this point added as stipulation or limitation?

**Authors’ response: This is an excellent point that we did consider addressing with a separate game. Unfortunately, only a small subset (n = 2) of subjects completed the “transfer” game at both time points (pre/post), which was too small of a sample to draw conclusions about the transfer. We have made an attempt to temper our conclusions about the clinical relevance of the cross-education of skill based on this game.**

3. Were participants screened for occupational, musical or sport training that could contribute to facilitation of the learning/practice effects reported in the study?

**Authors’ response: we used a convenience sample from the undergraduate population of the University of Victoria and participants were not screened based on occupational, musical or sport training.**

4. Line 190 – as stated, it isn’t clear how a ‘low-level wrist flexion contraction (10% of maximum)’ was measured/monitored? Was there a load cell built into the apparatus to measure maximum and steady-state wrist flexion torque? Or was this based on EMG amplitude? Please clarify.

**Authors’ response: Thanks for pointing out this omission of details. We have added that there was EMG feedback given to participants in the following way: “Feedback consisted of a 100 ms moving average of the rectified EMG.”**

5. The resolution of Figure 1-3 is low. It is difficult to see what is being depicted here. Some of the axis labels and in-figure labels are illegible. Please fix this.

**Authors’ response: We are not sure how this occurred, because on our end, the figures are high-resolution and very clear. We hope that the figures are clear on this second submission.**

6. Errors and time to completion were measured during training (as stated on line 161). Were the participants aware that training performance was being monitored and were they given feedback as to their performance outcome? If so, how frequent was the feedback? To what extent could the effects observed in the present study be attributable to augmented feedback due to knowledge of results during training rather than from the cross-education effect? This consideration may warrant comment in the Discussion.

**Authors’ response: As stated “Errors and completion time were only recorded in lab training sessions.” No feedback was provided to participants about the number of errors that they committed. In this way, participants could only understand the number of errors if they counted them in their own heads, which was not apparent based on the observations of our team of investigators. We have clarified this in the methods, but we do not believe that we should add anything on this point into the discussion.**

7. Figure 2A - Visual inspection of the data presented in this panel seems to indicate that time to completion for the untrained limb at Pre1, Pre2, and Pre3 is less variable and faster (generally speaking) than for the trained limb. For example, the slowest time to completion for the untrained limb at Pre1 looks to be approximately 100 seconds faster than the slowest time to completion for the trained limb. Pre2 and Pre3 seem to illustrate a similar pattern. The authors state that there is no statistically significant main effect of limb or limb x time interaction (limb x time) nor were there “significant differences between pre-test values, or between the trained and untrained limb” (Line 259). However, intuitively, one would expect that at baseline, time to completion should take longer with the untrained (non-dominant) limb. I suppose a rebuttal to this could have something to do with a speed/accuracy trade-off where participants could favour speed over accuracy. This position is somewhat supported by the Error data in panel B for Pre1; however, Pre2 and Pre3 don’t really follow. Can the authors provide an explanation for this seemingly counter-intuitive distribution of trained vs untrained outcomes at Baseline timepoints in Panels A and B?

**Authors’ response: This is a great observation, and at first glance is quite puzzling. Indeed, it would be strange if the non-dominant limb was better at gameplay than the dominant limb. To understand this seemingly strange observation, one must consider the order of task completion. In all cases, the participants played the game with the dominant (trained) limb before playing with the non-dominant (untrained) limb. We believe that this order of gameplay most likely explains the visual trend in the data for the non-dominant limb to be faster (albeit with more errors in Pre1) than the dominant limb.**

8. The sections in the Discussion interpreting the ‘Improvements with the trained limb’ and ‘Improvements with the untrained limb’ highlight differences in the rate and extent of improvement in both limbs in the present study in comparison to what has been reported in muscle power studies (Line 337, 340, 352-356). I think the Authors could elaborate on potential mechanisms for this as the muscle control element is the apparent novel contribution here. I appreciate that the section on spinal excitability (Line 366) is meant to describe a potential mechanism, but this doesn’t really differentiate between mechanisms for muscle power vs mechanisms for muscle control.

**Authors’ response: It is our belief that strength is a skill, too. However training for strength and training for muscular control differ in the skill that is being performed. With strength, plasticity occurs to increase the force generating capacity of a particular group of muscles in a particular direction. This can involve facilitation of agonist and synergist muscles, while inhibiting antagonists. On the other hand, training for muscular control essentially involves training inhibitory circuits to ensure fine control and smooth movements, while enhancing the specific motor plan for the skilled movement that is trained. We have expanded our discussion to point out these subtleties.**

Line 478 – Information for Reference 23 is incomplete.

**Authors’ response:** **Our apologies – this must have been an issue with our reference manager. The reference should be:**

**Manca A, Hortobágyi T, Carroll TJ, Enoka RM, Farthing JP, Gandevia SC, Kidgell DJ, Taylor JL, Deriu F. Contralateral Effects of Unilateral Strength and Skill Training: Modified Delphi Consensus to Establish Key Aspects of Cross-Education. Sports Med. 2021 Jan;51(1):11-20. doi: 10.1007/s40279-020-01377-7. PMID: 33175329; PMCID: PMC7806569.**

Line 502 – Reference 32 – is Volume/issue information available?

**Authors’ response: Our apologies, again – this must have been another issue with our reference manager. The reference should be:**

**Sun Y, Ledwell NMH, Boyd LA, Zehr EP. Unilateral wrist extension training after stroke improves strength and neural plasticity in both arms. Exp Brain Res. 2018 Jul;236(7):2009-2021. doi: 10.1007/s00221-018-5275-6. Epub 2018 May 5. PMID: 29730752.**
