# Supplementary material for: 1894 revisited: Cross-education of skilled muscular control in women and the importance of representation
Source: PLoS One. 2022 Mar 17;17(3):e0264686. doi: 10.1371/journal.pone.0264686 (PMC8929574; doi:10.1371/journal.pone.0264686)
Supplement: Attachment — Submitted filename: Responses to editor and reviewers.docx [file pone.0264686.s002.docx]

**Decision point #1: Reviewer 1 believes that FCR possibly does not play a role in game task, and requests evidence showing the role of FCR in the task provided to the participants.**

Comment from reviewer 1: The authors failed to provide a rationale for associating the results of H-reflex in FCR to behavioral results. The authors did not provide any information about the role FCR plays in the gameplay. My expectation is that in the gameplay the wrist movement is small and rather fixed. That is, the wrist muscles including FCR would not contribute much to skill acquisition/ fine motor control.

**Authors’ response: Although the direct role of FCR in gameplay may be limited, its role in stabilization and control of wrist movements is relevant to improving the number of errors. For instance, the FCR works in synergy with the ECR to produce radial deviation, and the FCR acts to stabilize and flex the wrist. To better understand the role of FCR in gameplay, one should consider gain control of the limb during a task being trained. If gain is high, small adjustments in neural drive would result in large adjustments in kinematics, potentially resulting in errors. On the contrary, if training results in reducing the gain of muscles acting at the wrist (e.g. the FCR muscle), adjustments in synaptic input (descending neural drive or sensory input as examples) would simply result in smaller deviations in the kinematics and, thus, reduced errors. Therefore, although not the primary muscle used during gameplay to make fine motor adjustments, the FCR is an appropriate muscle to act as a proxy for gain to arm muscles distal to the elbow. If the reviewer disagrees, we would be happy to revise the manuscript with their suggestion in mind.**

**In any case we have added some rationale for the choice of this muscle (lines 180-186) and emphasized this as a limitation to our study, which can be found in the “important considerations” section of the Discussion (lines 441-447).**

**Decision point #2: Reviewer 1 has also critiqued the overall experimental design, which the Academic Editor does not support too. Please provide clear justifications and/or describe them as the study limitations in the manuscript as well as in the response letter to the reviewer.**

Comment from reviewer 1: Both the time to completion and the number of errors continued to decrease (improve) in the three pre assessments (Fig. 2AB). In other words, repeated assessment improves behavioral performance. The fourth assessment was conducted after the training period, but it is possible that the same results would have been obtained if it had been conducted before the training period. It is a fatal problem that the three pre assessments did not provide reliable baseline.

**Authors’ response: This is an interesting point but we are also slightly confused. The reviewer seems to be arguing that the training exposures didn’t actually train the participants and that without the training a 4^th^ assessment would have yielded a training effect on its own. Is this correct?**

**Also, please note that when using statistical tests things are either significantly different or they are not. There were not significant changes across baseline tests according to statistical evaluations. Therefore the comment that measures “continued to decrease (improve)” is disingenuous. Even if this were true, and statistical testing supported the reviewer’s suggestion of perceived differences, this actually means we have underestimated the power of the training intervention and are actually being more conservative in our assessments. If the reviewer is asking us to say that our non-significant differences across baseline are “significant”, and we extend this logic, would we then be asked to do the same for any of our experimental measures?**

**In any case, we think we understand what the reviewer is getting at and have added “Although there were no significant changes across baseline measures, visual inspection of our data may suggest a trend towards improvement in these sessions. Future studies may consider multiple post-test measures as a way to evaluate this.” to the “important considerations” section (lines 422-425).**

**Decision point #3: Please respond to the minor comments of reviewer 3**

1. Regarding my previous comment about the counter-intuitive observations of the data presented in Figure 2A, it should be clarified in the Methods that participants always performed the muscular control assessments with the Trained hand first. As an aside, I wonder whether the Authors’ response and acknowledgement of Reviewer 1’s comment about startle in the Limitations section may also contribute to my point regarding Figure 2A. Performing the task with the Trained hand first and experiencing errors (i.e. buzzers sounds) may increase time to completion initially, but may then lead to an attenuation of the effect by the time participants performed the task with the Untrained hand.

**Authors’ response: We agree with both points, and as such have added a point in the methods (line 167) about the test order, and in the further considerations (lines 432-434) about how the habituation of the startle resulting from the buzzer may have contributed to the counter-intuitive results of the non-dominant hand completing the game at a faster rate.**

2. I note that References 32 and 33 are duplicates. Please correct.

**Authors’ response: We have revised by removing 33.**

3. The resolution of the images is still poor, but I this could just be on my end.

**Authors’ response: We apologize for this. The uploaded figures are clear on our end. We will ensure they are clear if the manuscript is accepted. We have uploaded .tif files exported at 300DPI from Adobe Illustrator, for the record.**
